# Building less-flawed metrics: Understanding and creating better measurement and incentive systems

**DOI:** 10.1016/j.patter.2023.100842

**Published:** 2023-10-13

**Authors:** David Manheim

**Affiliations:** 1 Technion - Israel Institute of Technology, Haifa, Israel; 2 Association for Long Term Existence and Resilience (ALTER), Rehovot, Israel

## Abstract

Metrics are useful for measuring systems and motivating behaviors in academia as well as in public policy, medicine, business, and other systems. Unfortunately, naive application of metrics to a system can distort the system and even undermine the original goal. There are two interrelated problems to overcome in building better metrics in academia and elsewhere. The first, specifying evaluable metrics that correspond to the goals, is well recognized but still often ignored. The second, minimizing perverse effects that undermine the metric or that enable people to game the rewards, is less recognized but is critical. This perspective discusses designing metrics, beginning with design considerations and processes; the presentation of specific strategies for mitigating perverse impacts, including secrecy, randomization, diversification, and post hoc specification; and continuing with important *desiderata* and tradeoffs involved with examples of how they can complement each other or differ. Finally, this perspective presents a comprehensive process integrating these ideas.

## The bigger picture

Metrics are a near-universal feature of the modern world, but they are often poorly suited to the tasks for which they are used. Perhaps most critically, systems are distorted by the metrics used so that people work toward the poorly designed metric in ways that contribute to neither their own goals nor the goals of the system designers. This perspective asserts that these problems can often be avoided and illustrates the considerations and key issues encountered when designing metrics.

Within academia, prominent scientific metrics exhibit many of these issues. In place of well-designed multidimensional measures, there is widespread reliance on one-size-fits-all metrics like the H-index or journal impact factors for hiring, tenure, and promotion. This collapses important multifaceted issues into metrics that undermine the features they intend to measure. For example, datasets and software are less citable, and less rewarded, than their importance deserves.

## Teaser

Design methods and consideration of *desiderata* for metrics have been proven useful when used, which is, at present, sporadically and inconsistently across a variety of fields. This perspective presents those methods and considerations in a single article. It then provides a clear methodology for designing metrics to avoid key problems, including insufficient connection between goals and metrics, and the perverse incentives metrics can create.

## What is the problem, exactly?

Metrics, key performance indicators (KPIs), targets, quantifiable goals, measurable results, and objective assessments are a few of the terms that get used to refer to the modern obsession with numerical and purportedly scientific ways to understand and measure human systems. From the perspective of a system designer, the use of measures as metrics has made evaluation possible and has led to improvements in business processes, medicine, education, and academia.

However, because of this success, and because of broad and sometimes unthinking overuse of metrics, there have been highly publicized failures, in the sense of sub-optimal outcomes for both designers and participants. Both Campbell and Goodhart identified an important failure mode for measurement, which was later paraphrased by Keith Hoskins as “every measure which becomes a target becomes a bad measure.”^1^^,^^2^^,^^3^ In other words, by transforming a measure into a metric, the measure changes. Campbell, who seems to have discovered the concept first, was a social scientist looking at how metrics distort behavior and saw that their use led participants to exploit metrics.^4^ Goodhart, on the other hand, was an economist noting a structural breakdown in inference about a system that occurs when rules change—a precursor to the now-famous Lucas critique in economics.

These challenges are also very predictable in the context of modern systems. For instance, academics have noted that higher education has fallen prey to “the tyranny of metrics”—a phenomenon that has been noted in various forms for decades.^5^^,^^6^ And the lesson has evidently not been learned in newer areas. Thomas and Uminsky note that it is a “fundamental challenge for AI,”^7^ and other research has laid out the distinct failure modes and underlying dynamics,^8^ specifically with reference to challenges in artificial intelligence (AI). The problem is no less severe when lives are directly on the line. In April 2020, it was clear how much reliance on poorly considered metrics would be deleterious for COVID-19 response,^9^ yet exactly those dynamics were observed in later studies.^10^ These are failures not just for the system designers but for everyone affected by the systems they design as well. That is, “gaming” metrics is not even beneficial for the participants.

And these failures now are well known across fields, from education,^11^ to warfare,^12^ to business.^13^^,^^14^ The failures occur when the measure is not aligned well with the true goal, when the system promotes cheating, that is, explicitly breaking rules, or when a less relevant or formerly useful measure is applied despite lacking current validity for the goal. And these failures are not just bad for the metric designer, as they can easily harm the participants as much or more.

Thankfully, more clearly understanding the failures and then looking at options that exist for metric design can point toward solutions for those in need of metrics. After reviewing those two topics, this perspective will consider *desiderata* that may be important in different domains and, finally, outline a better process.

## Delineating the problems with metrics

As analyzed in earlier work,^8^ there are distinct Goodhart-Campbell dynamics in using metrics to manage systems, each of which leads to predictable failures. Focusing on the failures rather than the mathematical dynamics, we note four high-level causes. The first is the difficulty of finding task-appropriate data, the second relates to imperfect correlation, the third is misusing correlations in ways that can cause perverse incentives, and the last is confusion about the goal to be measured or, relatedly but worse, fundamental incoherence.

In the first case, easy to measure is rarely the same as important,^15^ and easy to understand is not the same as relevant. Unfortunately, it is easier to collect data that is superficially related than to identify what is needed. In nutrition research, self-reported diet and energy intake is a relatively easy quantity to measure but is inaccurate^16^ and is obviously easy for a respondent to falsify. As another important example, J.E. Hirsch, a physicist, proposed “an easily computable index, h… of a scientist’s cumulative research contributions” that “can be found very easily by ordering papers by ‘times cited’ in the Thomson ISI Web of Science database.”^17^ Even aside from the issues with manipulation and unconscious biases created by the metric discussed below, the choice of metric was largely dictated by the availability of data. And as computerized systems and automated measurement have become common across fields, generated data have become easily available, but availability is often at best unrelated to usefulness for evaluation.

In the second case, a metric that is currently statistically correlated with the goal will inevitably be less closely correlated, for example, when conditioning on high values of the metric. As an intuitive example, height and basketball skill are correlated, but among the tallest people, it is unlikely that the best few basketball players are also the tallest. An additional common and well-understood problem is ignoring the difference between causation and correlation—a cardinal sin when attempting to improve a system. A closely related problem occurs when a metric is correlated with an intermediate measure or outcome, which itself correlates with a goal. For example, a sports team may use 100-meter race times as a metric for athleticism, but this only indirectly relates to success in the sport. This has the added issue of ignoring that correlation is not commutative. As an example of this non-commutativity of correlation (and as an example of divergence in extreme cases, discussed below), taller people are better at basketball, and coordinated people are better at basketball, but very tall people tend to be less well coordinated than moderately tall ones.^18^

In the third case, not only is a metric without a causal relationship invalid for evaluation and other purposes but using it can be pernicious. Validity of a measure is a critical ontological and epistemological necessity in research, and validity requires a causal relationship^19^ in the domain of interest. This is not simply the old saw about correlation being confused with causation, nor nitpicking limited to the philosophy of science. Two failure modes are due to the use of correlated metrics. The first is unwarranted extrapolation of correlation to extreme cases, as in the height example above. The second is the perverse effect of incentives that have an indirect relationship with the goal. Incentives for metrics that do not cause the eventual goal will, unsurprisingly, lead to pursuing the metric in ways that may not cause the goal. When explicitly optimizing a system using a metric, the optimization by one party changes the equilibrium response so that the metric becomes invalid because participants react to the new rules, as Campbell noted, and can even be actively harmful. If a teacher notes that students who ask questions learn more, they might announce that they will assign a portion of the grade based on the number of questions asked in class. The new incentives are likely to have the perverse effect of incentivizing questions, even those that detract from student learning. The metric would then not only fail to capture the important feature that allows learning but also carelessly harm the intended goal by encouraging misbehavior. Similarly, pushing for tier-1 publications means that people who want tenure are better off keeping data private for further publications than publishing datasets.

In the fourth and final case, the goal can be unknown, incoherent, or conflicting. The three cases above make an implicit assumption shared by both Goodhart and Campbell that the goal is both understood and coherent. A simple example of where this assumption fails is a committee composed of individuals with differing values and goals. If the differences are not understood, the goals are often incoherent. Even if they are understood, however, the individual goals may be diverse or even incompatible. If so, there may be no way to assign a coherent metric that will even correlate positively with all of them. For this reason, if the choice of metric is a compromise that does not address the conflict, the metric chosen and the resulting incentives may be incoherent. Similarly, the relationship between the goal and any measurement may be unclear. One common example is when the outcomes do not occur within a time frame that can be captured and when intermediate outcomes that can be measured have unknown or poorly understood relationships with the final goals.

An example of all of these issues in this final case occurs in the education system. The desired outcomes of education may include the student’s potential future academic contribution, their life satisfaction, their fitness for the job market, fostering their intellectual curiosity, and/or creating informed citizens. These are all hard to measure or discuss concretely, are not often discussed by those setting priorities, and are often conflicting with each other. Unsurprisingly, various intermediate metrics like grade point average (GPA) or college completion are poorly correlated with the desired long-term outcomes^11^—and the difference is subject to gamification—and the focus can have perverse effects.^20^ This is unsurprising. The degree to which incoherent, conflicting, or poorly defined goals can be achieved is intrinsically limited. Worse, as Deresiewicz argues,^14^^,^^21^ imposing simplistic metrics distorts education and defeats the original goals, and other approaches are needed.^22^

## Addressing the problem

The problem statements above help gesture toward solutions. Unfortunately, the solutions are not always simple or practical, and as this perspective will explore later, different approaches are viable and acceptable in different areas. Still, concrete examples of how the problems are typically addressed can show what viable and non-viable solutions look like.

To address the problem of collapsing correlation, it is often possible to build metrics that more closely relate to the actual goal. In our first example, instead of using conference attendance as a proxy for academic contribution, we can consider several outcomes, like conference invitations and paper acceptance, funding, time spent on research, and count of tier-1 publications. Combining these imperfect measures can at least mitigate some of the potential issues.

To address the second problem, metrics distorting the system, metric designers need a two-pronged approach. The first prong requires understanding how the measured quantity relates to or affects the goal. In the example, if the causal relationship between citations and academic importance is understood, evaluators will consider the determining factors of the relationship, such as citation context, and the diversity of citing authors and approaches. Thinking through these causes will hopefully make it clear that promoting more citations on its own will have an unclear effect. The second prong is ensuring that metrics are not being manipulated by the participants or at least minimizing this manipulation as discussed below, perhaps via secrecy, randomization, or post hoc choice of metrics. For example, if tenure applicants are unaware of which specific metrics will be used, their actions will less severely distort the metric—though there are obvious fairness and transparency concerns that will be explored.

In the third problem, clearly understanding the relationship between the data available and the goal can at least point to where issues can emerge. Sometimes, this leads to gathering different and more appropriate data. In the example of nutrition, scientists can measure actual food intake or the presence of micronutrients in individuals directly to reduce the number of steps between what they intend to measure and what the data provide. In the case of scholarly research, more careful attention to goals and the incentives created seems critical.

Lastly, incoherence and conflicting goals can sometimes be addressed with structured discussions leading to increased clarity. In such situations, compromise is often needed. Abandoning the search for an optimal solution or compromising on key goals may seem unfortunate, but the alternative of using incoherent metrics based on incompatible goals is often worse than doing nothing at all. Furthermore, where clarity and compromise are possible, coherent goals can be found. Compromising on goals and resulting metrics can lead to solutions that are acceptable to all participants. That is, they satisfice, in the terminology of the late, great Herbert Simon.^23^ Alternatively, complex approaches like robust decision-making deal with deep uncertainty and disputed values.^24^^,^^25^ Unfortunately, these methods usually cannot replace metrics and incentives, in part because they require far more difficult-to-understand methods, as well as needing analytic expertise and intense management involvement.

## Metrics and incentives across domains

Clearly, metrics, and the problems they create, are not limited to any specific domain, and the issues across domains will differ. The “scientific management” movement was an early proponent of reward systems like those used in corporations today: profit sharing, per-task payments or bonuses, and merit-based pay.^26^ In each case, the reward is tied to a metric: profit, tasks completed, and merit evaluations. On the other hand, motivation is complex, and there are important trade-offs between positive and negative reinforcement.^13^ These trade-offs are not just practical but also have ethical implications, leading for some to call for an ethics of quantification.^27^ For example, in the measurement of autonomous vehicles, a recent report suggested that the measures must be “valid, feasible, reliable, and non-manipulatable.”^28^

In addition to the cases above where at least a semblance of a metric is seen, the *desiderata* usually extend to motivation systems in general. For example, punishment systems have many similar features—law enforcement is less effective when arbitrary, when the punishments are often avoided, or when the perpetrators of what would normally be criminal acts find technical ways to avoid culpability. Prize competitions use measurement even more directly as a motivator, but participation will be limited if potential recipients worry about unfair treatment or corruption. Lack of clarity about goals, discussed above, would be even more critical when designing a direct incentive because without specification, the people being motivated will not understand the goal or be able to know when it has been accomplished. If it is instead specified clearly despite incoherence, rewards are likely to be either impossible to receive or trivially accomplished in ways unrelated to the goal.

## Strategies and trade-offs

The design of metrics requires an understanding of the design goals, the potential strategies available, and the trade-offs involved. To introduce these issues, we first outline useful *desiderata* for a metric. Following this, there are specific metric design considerations and strategies that involve the process of creating and considering the metric. These are not reflected in the metric itself but can lead to better choices of metric. Lastly, there is a set of metric features. Concluding the discussion of those features is a final point that not all problems can be effectively addressed using metrics. In such cases, rather than abandoning concrete numerical metrics altogether, we should start by reconceptualizing what they are being used for and how.

### Metric desiderata

Many properties of metrics exist in tension with one another.

Ideally, of course, we want metrics that give free, understandable, fair, incorruptible, and immediate insight. Unfortunately, we instead often get expensive, confusing, biased, unreliable, and out-of-date metrics that provide little insight. In addition to operational challenges like cost and availability, there are *desiderata* involved in choosing and using metrics for decision-making and incentives. The exact trade-offs between various motivational factors are a matter of intense empirical focus, and different domains have additional critical *desiderata*, but by stepping back from those discussions, we can see that those discussed below are often important.

Metrics generally benefit from (1) availability, (2) cost, (3) simplicity, (4) various forms of fairness, (5) trust, and (6) non-corruptibility. Specifically, availability, and immediacy, is useful for ensuring that feedback can be applied quickly and that participants can learn what is expected. For example, delayed rewards like end-of-year bonuses may be less effective motivators than immediate feedback. Simplicity is important in motivating behavior; complex metrics may be less effective motivators and may impose costs on both the participants and the evaluators. Fairness is also important for legal and social reasons, and even if an unfair metric is able to accomplish the intended narrow goals, it can lead to longer-term issues and undermine social trust. Trust enables positive interactions between participants and management and can help avoid or mitigate principle-agent problems. Insufficient transparency can also undermine perceptions of fairness and reduce trust, and transparency is sometimes a legal requirement. Corruption, of course, is a more direct attack on many of these *desiderata*, and either the perception or the reality of manipulation can do enough harm to more than outweigh any possible benefit from the use of a metric. More central to the problems of Goodhart’s and Campbell’s laws is that employees almost always analyze the system and sometimes intentionally or unintentionally are motivated to undermine goals.

Realistically, metric design needs to accommodate what is possible. Keeping the various *desiderata* in mind makes it possible to improve choice and design of metrics and incentives. The *desiderata*’s value must be weighed in each case against the costs, the importance of preventing gaming, and the impact of gathering the data. We therefore define the *desiderata* so that we can note where there are obvious advantages or conflicts that should be considered.

• Cost: Are the extant data free? Alternatively, how expensive is it to collect the data needed to compute the metric?
• Availability: Are the data needed to compute the metric available, or do they need to be collected? Are there lags in the process?
• Immediacy: Can the metric and/or incentive scheme provide feedback rapidly enough? Will lags in the system create instability or uncertainty?
• Simplicity: Is the metric easy or difficult to understand? Are the inputs to the metric understood? Are the implications of behavior clear? Will participants understand these factors well enough for rewards to influence their behavior and/or well enough to attempt to manipulate metrics? Will this change over time (in good or bad ways) as participants become accustomed to the system?
• Fairness: Is the metric commensurate to actual goals? Does the metric provide disproportionate benefit to some groups? Do behaviors that get influenced by the metric impose costs elsewhere in the system?
• Trust: Do administrators and participants trust one another not to manipulate the metric? Can manipulation be observed by both parties? Will participants and administrators trust the system or the transparency measures enough to believe that it is not being manipulated?
• Non-corruptibility: Who has access or ability to change the data or manipulate them? Does the metric introduce exploitable information asymmetries? Can the system be used by participants to cheat, and is such cheating incentivized? Can it be manipulated by administrators?

### Design considerations

In light of the challenges discussed at the beginning of this perspective, and the *desiderata* listed above, we suggest five general thought processes and factors to consider that can be useful in designing better metrics, with a focus on avoiding metric over-optimization failures and corruption.

• Coherence: If the goals of a system are incoherent, or are poorly understood, it will be difficult for any metric to capture them. For example, it is easier to measure lines of code written by a programmer than it is to judge how well the code performs. In some cases, the metrics in place serve simply to justify the status quo or to act as window dressing. Promotions in companies may in theory be based on metrics, but if managers can choose to apply the metrics selectively, this can serve as a mask for justifying decisions made on a different basis.
There is a common temptation, in part driven by cost, to find easy-to-measure outcomes instead of choosing based on how well a measure represents the goals or based on the value of better data.^15^ Unfortunately, this temptation is too-often yielded to in practice, either due to lack of thought or too little consideration of the impacts of poorly built metrics. This is especially common given incoherent (or under-specified) goals, where the fuzziness leads to losing sight of the purpose, and not measuring what is important to the process.^29^ This confusion is a key cause of strategy surrogation, where managers forget that measures are imperfect proxies and improperly reify the measures as identical to their goals.^30^
• Causal forethought: Sometimes, the metric measures something related to the intended goal with an unclear or non-causal relationship. If this is the case, a reward system using that metric can creates incentives that make the relationship between the metric and the goal disappear. For example, measuring attendance in class may increase attendance, but if the otherwise-absent attendees spend their time in class sleeping, or being disruptive, it is possible that nothing will be gained. A theory of change is helpful for clarifying these relationships and avoiding this class of error. (See Taplin and Clark’s book for a clear introduction to theory of change.^31^)
• Structured discussions and compromise: In situations of deep uncertainty and conflicting goals, there is often a need for discussion and compromise. While no compromise can achieve conflicting goals, deep exploration of problems can often lead to agreements that are better for all participants than the alternatives.^25^^,^^32^ While useful, these methods require extensive and costly analysis and discussion and are therefore ill-suited to many smaller-scale problems.
• Pre-gaming: If a metric is proposed, the exercise of imagining how it could be gamed, and building incentives aimed at forestalling gaming, can be useful. This idea is closely related to research about the effectiveness of such planning by Mitchell, Russo, and Pennington,^33^ which Gary Klein later popularized as a pre-mortem.^34^ If done well, these can be very helpful—but some care should be taken about how to actually run such exercises, as they are often carried out poorly.^35^ After identifying likely failure modes, it may be possible to improve the metric or to add explicit conditions to the rewards to thwart the failure modes that were discovered. Despite the desire to restrain gaming, however, care should be taken to ensure that the metric does not dictate exact methods, which can stifle innovation in favor of accomplishing the overall goal. For example, measuring hours of classroom time spent by a teacher may discourage time spent on lesson planning, peer consultation, and other activities that improve effectiveness of the time spent in class. Explicitly requiring each of those specific activities to account for the potential failure, however, removes discretion that allows teachers to pick the activities that are most beneficial in their case.
• Monitoring behaviors: Even when well designed and initially effective, metrics tend to go awry over time as systems and behaviors change. Explicitly setting checkpoints and reviews for metrics may be useful for ensuring that these systemic drifts are limited in scope. This is especially useful when it is easy to detect cheating. For example, metrics often promote a short-term, intermediate goal, like sales of a certain product or short-term ad revenue. Incentives may start encouraging overzealous sales activities or placement of ads that interfere with user happiness or engagement, in each case potentially preventing longer-term growth. Overzealous sales activities would be visible in lower repeat sales or reduced customer satisfaction, so monitoring these might detect the failure early. Designing perfectly coherent metrics aligned with goals for the system overall may be infeasible, but monitoring behavior can help detect or prevent larger distortions and later systemic failures.

To conclude the discussion of design, there are often trade-offs between ease of measurement, cost of measurement, and the better solutions that can result from the above processes. This means that the time invested in metric design should be commensurate with the importance of the metric, the potential impacts, and the likelihood of manipulation or perverse effects. Sometimes these issues are minimal, and ease of measurement is paramount. Still, the choice of easy or convenient metrics should be intentional rather than a default caused by ignorance of the potential issues.

### Metric features

The *desiderata* are difficult to balance, and the processes suggested can clarify goals and weaknesses of a design. In addition, there are specific features of metrics that can allow for different and sometimes better trade-offs. Hopefully, the below partial list of metric properties, along with suggestions about strategies and avoiding perverse outcomes, is helpful.

• Data sources: There are many places that can be used for understanding a system, and not all of them are immediately obvious to metric designers. For example, in medicine, administrative data can sometimes be as useful as clinical data for understanding risk but do not need to be gathered separately.^36^ Similarly, for websites, user behavior can be gathered from site logs and used to infer issues rather than fielding user surveys to ask about the experience. Of course, it is important to again caution that easy to measure is rarely the same as important.^15^
• Diversification: Often, no single measure can be found that aligns well with the goal and that is not manipulable. Introducing additional measures into a metric, even if they are individually less well correlated to the goal, can sometimes improve the system overall. In a similar way, multiple different metrics can align with the true goal than any single metric.
• Aggregation: Diverse and compound metrics can be used to mitigate problems with incoherence, such as disagreement or lack of causal understanding. This is because a scattershot approach will tend to limit the degree to which any one measure influences the system. Designers with conflicting goals can choose measures that assist with each, and the combination may be an acceptable compromise. Similarly, if the causal relationships are unclear, targeting multiple different parts of the system may constrain the extent of failures due to the new incentives.
• Secret metrics: If the metric is not known to participants, they cannot game it. The existence of an unrevealed metric can still incentivize participants to achieve the goals they think most likely to be measured or rewarded, and to the extent that they understand the goal but not the metric, this will align incentives while hindering manipulation.
• Post hoc specification: If the metric is chosen after all actions are taken, participants view the metric as secret, but because the order of choices is reversed, attempted gaming of the metric can be punished or at least detected and ignored. Unfortunately, this can be perceived as allowing unfair discretion and may lead to new forms of corruption in both the technical sense of invalidating the metric and the typical sense of dishonest and fraudulent conduct by those choosing the metric.
• Randomization: Even if a metric is known beforehand, if the relative weights or reward formulas are uncertain, gaming the metric is harder and, in expectation, less rewarding. In addition, many forms of randomization can assist evaluation of success via econometric methods or monitoring the usefulness of the metric. Again, however, this reduces perceived fairness.
• Soft metrics: Human judgment, peer evaluation, and other techniques may be able to reduce manipulation. Metrics are often seen as a way to avoid subjectivity, but a combination of quantitative measures and human judgment can capture the best of both worlds.
• Limiting maximization: Failures are often the result of too much optimization pressure. Using metrics to set a standard or provide a limited incentive, instead of a value to maximize, can mitigate these failures.
• Abandoning measurement: Sometimes, the best solution is to do nothing—or at least nothing involving measurement—and avoid what Muller refers to as metric fixation.^5^ Metrics should not be used if the value of better incentivizing participants is lower than the impact of perverse incentives.

## Applications and metric features in practice

Not all strategies are appropriate in all domains, and implementation is critically dependent on factors specific to a given system and the relevant actors. Still, systems chosen by public authorities face a higher burden for fairness and non-corruptibility, while those implemented in private business often require more immediacy. Incentives intended to motivate non-experts benefit more if they are simpler and easily understood, and those that impact people or organizations that must participate in a system, such as employees, or those that involve high reward may need to be more game-proof.

The different issues that are implicated necessitate a broader discussion of some of the complex trade-offs. The variety of concerns that exist, however, make it worthwhile to illustrate the relationship between the metric *desiderata* and the process of designing better metrics and how the different specific metric strategies will affect the *desiderata*. Table 1 attempts to do this briefly, followed by a discussion of how *desiderata* can differ based on more specific context.

**Table 1:** Desiderata

	Availability	Cost	Immediacy	Simplicity	Fairness	Non-corruptibility
Considering coherence		+		#	+	+
Causal analysis		–		–		
Structured compromise				–	+	
Pre-gaming		–			+	+
Monitoring behavior	–	–	–	#	+	+
Diversification		–		–	+	
Aggregation				–	+	+
Secret metrics			–		#	–
Post hoc specification	+	+	–			–
Randomization			#	–	–	+
Soft metrics	+	–	#	#		
Limiting maximization				–	+	
Abandoning measurement			+	+	–	–

The table indicates which *desiderata* are likely affected by which strategy and metric characteristic. Positive effects on each *desideratum* are indicated with a plus, while negative ones are indicated with a minus. Complex interactions are noted with a hash, as these are sometimes positive and sometimes negative.

There are many examples of considering these and related *desiderata*. Reviewing a few recent, exemplary examples allows us to highlight how context-specific features lead to *desiderata* and approaches that are unique to that context.

Fraade-Blanar et al.’s “Measuring Automated Vehicle Safety”^28^ usefully distinguishes between “measures (concepts),” which can be thought of as soft metrics, and “metrics (a defined calculation).” In this framing, they note that measures can be leading or lagging so that the leading measures are indications, typically without a clear causal relationship with the goal, which “serve as proxies or surrogates for lagging measures,” which may come too late but can be more precise and causally connected to the goal. They suggest that the measures should be valid, reliable, feasible (low cost), and non-manipulatable (non-corruptible).^28^ Because they focus on leading indicators, the discussion of validity drops their earlier and critical discussion of how measures should have causal, in this case physics-based, relationships with the phenomenon of interest. Reliability is important in their context because the metrics are used across all vehicles and vehicle types, and measures may differ in their validity or usefulness between vehicles.

O’Keefe et al.’s Windfall Clause discusses designing a quantifiable future trigger for companies that capture large windfall profits from being the first to invent general AI and considers *desiderata* that include transparency (trust) elasticity and adequacy (fairness) and a number of less generally applicable *desiderata*.^37^ The less applicable *desiderata* here are interesting because of the speculative nature of the metrics—there is no way to validate them before the potential one-time event they are supposed to influence.

Development impact bonds are an application of metrics that faces many challenges due to being directly financially incentivized. In addition, they need a metrics specified in advance that resolves quickly at the time of the bond maturity, so groups designing such bonds must be very careful. Sturla, Shah, and McManus^38^ present a very useful summary of the lessons learned by IDInsight in this domain. First, they need to measure carefully, using “outcomes that: 1) capture real improvements in people’s lives, 2) can be measured, and 3) hold up under pressure.” Second, the impact must be accurately and convincingly attributed, implicating both trust and transparency. In this case, attribution also requires careful understanding of the causal basis of the measurement. Third, the goals need to allow for discretion in implementation and allow adaptation during the process so that innovation is possible. Fourth and finally, design needs to carefully consider trade-offs, especially because these bonds are ideally designed so that the measurement can be done at low cost.^38^

In contrast to these positive examples, a single negative example will perhaps illustrate even more clearly the benefits of considering *desiderata* and finding metrics that are fit for purpose. The example, noted earlier for using easily available data, is the H-index, “a useful index to characterize the scientific output of a researcher.”^17^ And scientists have bemoaned its inadequacy almost since its proposal.^39^

Of course, “scientific output” is woefully underspecified and essentially incoherent as a goal. Despite this, the H-index and similar simplistic metrics like impact factors are now used widely, for tenure decisions, as a shorthand way of comparing eminent and less eminent scientists, for grant funding, and wherever else, as Hirsch concludes, evaluators wish to “compare, in an unbiased way, different individuals.” However, even before Hirsch’s index, others pointed out that for academic metrics, “each type of indicator reflects a particular dimension of the general concept of research performance … [and] one single indicator only may provide an incomplete picture.”^40^ It was already clear why any “universal metric” in academia would fail—and this issue is greatly worsened by the perverse incentives created, a problem entirely unmentioned by Hirsch.

Given these concrete examples, it is now worth considering what things should be considered for building and calculating metrics and how they can help.

### Data sources

New or unexploited sources of data can be very valuable. Often, new sources are marginally the most valuable sources for metrics because they provide novel insights.^15^ At the very least, the novelty itself can temporarily forestall gaming the metrics. At the same time, new instruments and data sources will have new and unforeseen challenges, and the ways in which they fail can be far less obvious. One important question is whether the data are open and whether the benefits of increased transparency outweigh the costs of making metric manipulation easier and enabling questionable research practices such as HARKing.

### Diversification

When goals are complex but cannot be directly measured, measures of various components or correlated outcomes can be used. Multiple measures can also help “triangulate” the goal that cannot be easily found with a single measure.^41^ This may make the goal easier to achieve since it replaces an unclear target with clear sub-targets, but it may also make it harder for participants to decide what they should focus on, increasing complexity and confusion. The strategy can also make gaming of metric harder, but each additional measures also creates the need for the evaluator to identify how it can be gamed and how to prevent that gaming.

The strategy is often not only helpful but necessary. When a metric includes only some parts of a goal, it implicitly pushes emphasis away from the others. Diversified metrics can mitigate this issue. If reading and arithmetic are each 50% of the measured outcomes from school, science, art, and physical education are all 0%. Because the measured parts of a system are optimized for, even rudimentary or biased measures of the remaining outcomes can reduce bias.^15^ That is, measuring additional features removes the implicit pressure to minimize the previously unmeasured parts of the goal. For example, adding measures of time spent in arts classes will at least mitigate the pressure to remove those classes completely—and by doing so, lose important longer-term benefits that are more difficult to measure for short-term evaluation.^42^

Note, however, that simply adding metrics may not be wise, especially if they are all capable of being exploited in the same way. For example, testing students on various subjects to diversify metrics for learning instead of focusing on just mathematics and language does nothing to prevent metric failure if students cheat on tests. In addition, it may aggravate losing class time due to testing and teaching to the test.

### Aggregation

Metrics that amalgamate multiple simple measures are often useful when individual measures are insufficient, as noted in the discussion on diversification. Recalling an example used above, the choice of the best basketball players is better predicted by a combination of metrics than any single one. Aggregation can be used to side step issues with finding a consensus for a single metric. Aggregation can also be useful when causal relationships are unclear. In either of these cases, however, the metrics are unlikely to be coherent. Still, because the different metrics typically require different behaviors, and they will be, to some extent, in tension with one another, they can make gaming harder.

Note that diversification and aggregation can be complementary, but diversification does not require a single aggregate metric. Triangulation, for example, is made harder by aggregating metrics. Leaving multiple metrics disaggregated can also identify and prevent problems caused by Simpson’s paradox. Comparing sub-group outcomes directly reduces the incoherence of comparing implicitly aggregated outcomes. For example, Leibowitz and Kelley show examples where different sub-population sizes can make ranked education outcomes reverse direction markedly. Once the success of sub-groups is considered, diverse areas that perform worse in the aggregate are found to better serve every sub-population, making the aggregate metric for success not only incomplete but deleterious.^43^

There are benefits and costs to aggregation. Aggregation can make it easier to evaluate, compare, or incentivize results. On the other hand, combining conflicting or varied measures will make the overall system more complex and will sometimes make the goal incoherent. These are trade-offs. The complexity of aggregate metrics can also sometimes reduce the degree to which participants can game metrics but simultaneously make it harder for the designers to identify ways that participants may find to game the system, and complex or incoherent metrics are less effective at motivating behavior.

### Secret metrics

When the qualitative goals are understood, participant knowledge of the metrics may be unnecessary, and keeping participants from knowing the details of the measurement system will limit exploitation. The obvious example is testing, where the questions are secret. If, however, the qualitative goal is poorly understood, such as if the subject of a test is unknown, the participants will not be motivated.

There are several requirements for secrecy to work. If the goal is clear, then if pre-gaming methods discover important vulnerabilities of the various metrics that are hard to avoid and the metrics that would be used are not obvious to the participants, secrecy can be an effective strategy for preventing gaming. Alternatively, if the intent is to measure rather than motivate, especially if the measurements need not be provided to the participants, secrecy can be useful.

Unfortunately, even where it is useful, secrecy is prone to degrade over time either as rewards are received or as people infer what is being evaluated. If a metric must be used for feedback repeatedly or in real time, it will be difficult to keep participants unaware. Similarly, if managers or regulators who implement the system are themselves being judged based on the results, or if they can be induced by participants to cheat, a new failure mode is created. For this reason, secret metrics are more helpful if used one time and then changed (e.g., new tests are written for students each year).

### Post hoc specification

When results are seen and analyzed before the metric is chosen, there are a variety of ways to prevent gaming while preserving the transparency of the rewards. Post hoc choices can invalidate evaluation, especially if arbitrary. On the other hand, post hoc specification can keep the measures easy to understand.

Designing measures post hoc needs particular care to avoid justifying intuition or decisions already made. One solution is for post hoc specification to be limited to only including or excluding parts of a pre-determined aggregate metric. Alternatively, only the weights on various measures may be chosen post hoc, or certain measures may be discarded based on analysis of the outcomes. If the process is known by participants beforehand, trust will be easier. Even better, the potential for metrics to be discarded or given low weights can serve as an incentive not to game them.

The first and most significant disadvantage for such post hoc decisions is unfairness, both actual and perceived. Transparency in the process for the post hoc selection can enhance trust, as can ensuring that the decision is made by an uninvolved third party. The second significant disadvantage is that the feedback and reinforcement is delayed, which reduces its effectiveness.

### Randomization

Randomization can be used to choose between different proposed metrics when there is disagreement or can be used within the metric itself. Allowing part of a metric or incentive to be determined by chance can be useful for preventing exploitation. Like secrecy and post hoc specification, randomization reduces the direct connection between behaviors and metrics, which has some of the same positive and negative impacts.

To the extent that the weights and rewards are randomized instead of chosen intentionally, the incentives will be less well aligned with the actual goal. The uncertainty may also be perceived as adding significant complexity, and again reduce motivation to achieve goals, but exploitation is similarly less rewarding. Randomization can also be perceived as unfair, either because it rewards individuals differently or because rewards are not proportionate to importance.

Randomization works well in combination with other methods. For instance, the randomization of the outcomes of a metric based on diverse inputs might assign random weights to known components. Similarly, it can be used to remove concerns about corruption for post hoc specification by pre-specifying the randomization to be performed. Alternatively, if used beforehand to assign different metrics or different weights on metrics to different groups, it can also be valuable for analysis and comparison of metrics and incentive systems.

### Soft metrics

Metrics can include quantitative evaluations of factors that require subjective evaluation. What Gottleib and Schneider^22^ called “Squishy” measures avoid certain pitfalls of focusing on quantifying extant data. For example, peer ratings by programmers will not reward behavior such as rapid but sloppy development, which impose costs to the overall goals and maintainability of a system. Such measures have their own potential for exploitation, where participants game the system via “sucking up” or by trying to appear productive instead of actually working.

Qualitative data gathering can be done routinely, providing feedback rapidly, but if participants spend otherwise productive time doing evaluations, then the cost of such measurement is high. They can also be perceived as unfair and lead to fighting or backstabbing—especially if the rewards are zero sum.

### Limiting maximization

Metrics do not need to be maximized to be effective. By replacing optimization with what Simon terms satisficing,^44^ finding a solution that achieves a fixed goal rather than maximizing, many problems can be avoided. For example, bonuses for salespeople who hit sales-number targets are less likely to lead to tactics where employees try to “steal” credit or alienate customers with overly aggressive tactics.

This strategy is not always appropriate, and using metrics in this way will not avoid gaming metrics to avoid underperformance, nor will it necessarily eliminate pressure to cheat.

Fixed goals instead of metrics can also create problems. Steven Shorrock noted that “when you put a limit on a measure, if that measure relates to efficiency, the limit will be used as a target.^45^” The original example there was of flight duty times, where a regulation limits the maximum number of duty hours that airlines crews can work. Once airlines were required to log crew-duty times, they tried to ensure that their employees are as close to the limit as possible. By introducing this new measure, it is possible that crews are now more overworked than they were before measurement of duty hours began.

Once measures exist, they will often be used as metrics even when inappropriate. As an example, the UK has a “Year 1 Phonics Check” in schools, which was developed and validated for diagnosing “at-risk-readers.^46^” From there, it quickly turned into an “accountability agenda.” Instead of diagnosis, it was repurposed for grading teachers’ and schools’ success at teaching reading.^47^

Satisficing can also allow complacency once targets are reached. Some climate legislation limiting emissions failed because they were not ambitious enough, and “the shortcomings identified … are inherent to crediting mechanisms in general.^48^” The report found that transferable emissions credits were worthless in part because there were too many credits that were being generated. This was made worse because of the ability to transfer the credits from countries that exceeded the goal to places where the goal was not met. Because no further incentive was in place once targets were met, there was no need for more ambitious projects. In such a case, structuring the incentive as a metric instead of a goal might have been more effective. For instance, a tax on emissions provides incentives for emissions reduction without providing a potentially unlimited incentive to artificially game the system the way refundable tax credits might.

### Abandoning metrics or sticking with measures

Despite their general usefulness, metrics are sometime bad, for instance, in situations where measuring outcomes is too expensive to be justified by the potential improvement that it could create. This can occur when the complexity of properly rewarding participants requires a business structure that is inefficient.^49^ In other cases, the metric distorts incentives more than it promotes the goal. The aphorism that what is not measured is not managed is correct. Still, when choosing between not managing part of a system by not measuring it or measuring it in a way that makes outcomes worse, the decision should be clear.

The negative impacts of the failure of metrics are felt by multiple parties. Obviously, the metrics designers would prefer if their goal was pursued rather than participants chasing the metric. Further, participants who attempt to target the goals of the system and ignore the perverse incentives are implicitly punished for not playing these games. The participants who adopt strategies to exploit the perverse incentives may benefit directly but would often be happier not to be forced to play the game of understanding and exploiting complex, changing, and often harmful systems. And exploitation and failure of metrics often has broader impacts, including significant economic waste and negative externalities created by exploiting poorly designed metrics.

### The gravity of abandoning metrics and the alternative

Choosing not to manage a system is a decision that should not be made lightly. On the other hand, putting in place a mediocre measurement system prematurely is often far worse than abandoning metrics. Until serious consideration has been given to the processes and alternatives identified above, it may be better to wait to build metrics, or to abandon incentives based on measurement, rather than deploy a system that will be ineffective or worse. As Muller puts it, “sometimes, recognizing the limits of the possible is the beginning of wisdom. Not all problems are soluble, and even fewer are soluble by metrics.”^15^

These limits Muller notes are particularly relevant if explicit rewards attract participants less well suited to accomplishing the goal than those who would participate regardless. The limitations are also critical if participants feel discouraged by extrinsic motivation and measurement, especially in the many domains where intrinsic motivation is primary. This is supported by the empirical work by Rasul et al.^50^^,^^51^^,^^52^ showing that autonomy, which is incompatible with extensive measurement and accountability systems, is more effective for civil service.

However, it is critical not to throw out the measurement baby with the perverse incentives bathwater. In most cases, measurements can be used for evaluation as a feedback mechanism rather than using them as metrics or a direct reward system. Measurement alone is particularly useful when qualitative feedback and supervision are useful. For example, instead of using metrics to determine who gets a year-end bonus, the same measures of performance might be used to identify which people are excelling and which are falling behind so that the former can mentor the latter.

Transitioning to monitoring via measurement is also very useful if the diagnostic measures cannot identify what is failing or if they are known to be causally distant from the goal. Identification of an issue can be useful without diagnosis, much like noise from a car engine is (usually) of limited value in diagnosing a problem but is of immense value in noticing that some such problem exists. In systems that are poorly understood quantitatively, diagnosing issues might require intensive investigation and intervention, but some numeric measures can provide early warning of a problem.

Still, as discussed above when considering limiting maximization, there are many problems that occur simply because measurements or concrete criteria are introduced. In addition to the above concerns, the use of quantifiable guideposts adds new failure modes. For example, these can be used to make claims unrelated to the purpose of the measurement, as in the earlier example of phonics testing, or can be used by other parties to accomplish other goals, sometimes undermining the purpose of the diagnostic measure.

As an example of how diagnostic measures intended for evaluation can be misused, consider diagnostic criteria in mental health. Used properly, criteria are interpreted with contextual factors, the presence or absence of extrinsic causes, the existence or non-existence of a functional impairment, and so on. These diagnostic criteria are intended to be flexible and provide insight and assistance for clinical work. “A too-rigid categorical system does not capture clinical experience,” but it is all too easy for non-experts (or experts) to use the diagnostic criteria far outside of what the criteria writers intended.^53^ In extrema, this leads to “amateur, at-a-distance diagnosticians” applying such criteria, even if correctly, as a political statement rather than for treatment.^54^

The same domain also illustrates the abuse of diagnostic criteria to accomplish other goals. Mental health diagnoses are used by American insurance companies to determine whether to reimburse treatments or how much to pay for a given service. In doing so, assessments can be turned into games played by clinicians (or their billing departments) to enable individuals to get needed care. This turns diagnostic measures back into metrics, with the accompanying failure modes. For example, diagnostic accuracy may be replaced with practical concerns. An insurance provider may not pay for counseling or medication in the case of a generalized anxiety disorder but the service or medication is covered if the patient is instead diagnosed with panic disorder. If a patient cannot otherwise afford care, the temptation for providers to modify patient diagnoses may be overwhelming.

### Toward a coherent process for metric design

Given the various strategies and considerations discussed in the paper, as well as failure modes and limitations, it is useful to lay out a simple and coherent outline of a process for metric design. While this will by necessity be far from complete and will include items that may not be relevant for a particular application, Table 2 should provide at least an outline that can be adapted to various metric design processes. Outside of the specific issues discussed earlier, there is a wide breadth of expertise and understanding that may be needed for metric design. Citations in Table 2 also provide a variety of resources for at least introductory further reading on those topics.

**Table 2:** Design process

1. Understand the system being measured, including both technical^55^ and organizational^20^ considerations.		
Determine scope	What is included in the system? What will the metrics be used for?	
Understand the causal structure of the system	What is the logic model or theory?^56^ Is there formal analysis^57^ or expert opinion^58^ that can inform this?	
Identify stakeholders^59^	Who will be affected? Who will use the metrics? Whose goals are relevant?	
2. Identify the goals	What immediate goals are being served by the metric(s)? How are individual impacts related to performance more broadly?^60^ What longer-term or broader goals are implicated?	
3. Identify relevant *desiderata*	• Availability • Cost • Immediacy • Simplicity	• Trust • Fairness • Non-corruptibility
4. Brainstorm potential measures for metrics	What outcomes are important to capture? What data sources exist? What methods can be used to capture additional data? What measurements are easy to capture? What is the relationship between the measurements and the outcomes? What is not captured by the measurements?	
5. Consider and plan	Understand how measurements will be used to build metrics.^61^ Consider how the metrics will be used to diagnose issues or incentivize people.^30^ Consider the use of soft metrics to triangulate.^41^ Consider avoiding the “reward/punish” dichotomy.^62^ Identify and mitigate likely failure modes with pre-mortems.^34^	
6. Plan to revisit the metrics	Set a specific date or interval for routine re-evaluation. Identify additional triggers for non-routine re-evaluation.	

## Conclusion

Despite the intrinsic limitations of metrics, the frequent use and evaluation of poorly thought-out and badly constructed metrics do not imply that metrics are doomed to eventually fail, or that evaluation should not be used because measures will be exploited. Instead, it seems clear that forethought and consideration of the problems with metrics is necessary to ensure valid measurement and non-harmful evaluation. This process starts by identifying and agreeing on coherent goals, then considering both what leads to the goals, and what parts of the system can be measured. After identifying measurable parts of the system and considering how participant behavior might exploit the measurement methods or the measured outcomes, evaluable measures can be constructed. The construction of these metrics to avoid exploitation may involve multiple diverse measures, secret metrics, intentional reliance on post-hoc specification of details, and randomization. This may also include decisions about where subjective measurements are important, and consideration of whether measurement will be beneficial. In building the metrics and deciding whether to implement them, attention should be paid to various important factors in the system, including immediacy of feedback, simplicity, and understandability of the measurement system, fairness, and the potential for both actual and appearance of corruption in the metric and reward system.

Metric design is an engineering problem, and good solutions involve both science and art. Following these guidelines will not make metrics impossible to exploit, nor will it keep everyone happy with the results of a process. This is true of metrics used for employees, metrics used for monitoring systems, and even metrics used within machine learning algorithms - in each case, poorly designed metrics will be exploited. Occasionally, the process suggested here will lead to an investigation of potential improvements. Other times, it will identify strategies that are ultimately decided against - but it is still a vast improvement on the too-common strategy of using whatever metric seems at first glance to be possible to evaluate, or building interventions or systems around metrics without considering what they in fact promote. Putting in the effort to build elegant and efficient solutions will not fix every problem, but it will lead to less flawed metrics and better results overall.
